# A Unique Pattern of Mesothelial-Mesenchymal Transition Induced in the Normal Peritoneal Mesothelium by High-Grade Serous Ovarian Cancer

**DOI:** 10.3390/cancers11050662

**Published:** 2019-05-13

**Authors:** Martyna Pakuła, Paweł Uruski, Arkadiusz Niklas, Aldona Woźniak, Dariusz Szpurek, Andrzej Tykarski, Justyna Mikuła-Pietrasik, Krzysztof Książek

**Affiliations:** 1 Department of Hypertensiology, Angiology and Internal Medicine, Poznan University of Medical Sciences, Długa 1/2 Str., 61-848 Poznan, Poland; mpakula@ump.edu.pl (M.P.); puruski@ump.edu.pl (P.U.); aniklas@mp.pl (A.N.); tykarski@o2.pl (A.T.); jmikula@ump.edu.pl (J.M.-P.); kksiazek@ump.edu.pl (K.K.); 2 Department of Clinical Pathology, Poznan University of Medical Sciences, Przybyszewskiego 49 Str., 60-355 Poznan, Poland; aldona.wozniak@wp.pl; 3 Division of Gynecological Surgery, Poznan University of Medical Sciences, Polna 33 Str, 60-535 Poznan, Poland; dszpurek@gmail.com

**Keywords:** cancer metastases, mesothelial cells, mesothelial-mesenchymal transition, ovarian cancer, peritoneal cavity

## Abstract

The study was designed to establish whether high aggressiveness of high-grade serous ovarian cancer cells (HGSOCs), which display rapid growth, advanced stage at diagnosis and the highest mortality among all epithelial ovarian cancer histotypes, may be linked with a specific pattern of mesothelial-mesenchymal transition (MMT) elicited by these cells in normal peritoneal mesothelial cells (PMCs). Experiments were performed on primary PMCs, stable and primary ovarian cancer cells, tumors from patients with ovarian cancer, and laboratory animals. Results of in vitro and in vivo tests showed that MMT triggered by HGSOCs (primary cells and OVCAR-3 line) is far more pronounced than the process evoked by cells representing less aggressive ovarian cancer histotypes (A2780, SKOV-3). Mechanistically, HGSOCs induce MMT via Smad 2/3, ILK, TGF-β1, HGF, and IGF-1, whereas A2780 and SKOV-3 cells via exclusively Smad 2/3 and HGF. The conditioned medium from PMCs undergoing MMT promoted the progression of cancer cells and the effects exerted by the cells triggered to undergo MMT by the HGSOCs were significantly stronger than those related to the activity of their less aggressive counterparts. Our findings indicate that MMT in PMCs provoked by HGSOCs is stronger, proceeds via different mechanisms and has more procancerous characteristics than MMT provoked by less aggressive cancer histotypes, which may at least partly explain high aggressiveness of HGSOCs.

## 1. Introduction

One of the most important processes involved in the progression of primary and metastatic tumors is epithelial-mesenchymal transition (EMT). This term refers to a process in which cancer cells that typically display a uniform epithelial morphology become spindle-shaped and more invasive. From the molecular perspective, EMT is linked with the repression of the epithelial markers E-cadherin, cytokeratins, and occludin and the increase in the mesenchymal proteins, N-cadherin and vimentin. These changes are regulated by a plethora of signaling molecules, of which Smad, Snail, Slug, miR-200, and TGF-β1 are the most important [1].

In accordance with Padget’s “seed and soil” theory of carcinogenesis, epithelial ovarian cancer cells (EOCs) display a preference for the peritoneal cavity as a site of metastasis. The intraperitoneal spread of the disease is driven by an interplay between the cancer cells and peritoneal mesothelium (PMCs). In general, the PMCs create a metastatic niche by supporting the critical elements of cancer cell expansion, such as adhesion, proliferation, migration, and neoangiogenesis [2].

The pathogenesis of the intraperitoneal dissemination of ovarian cancer also involves EMT. It has been recognized that the exfoliation and shedding of cancer cells from a primary lesion is linked with the declined expression of E-cadherin [3]. This state also characterizes free-floating cancer cells during their transfer by the peritoneal fluid [4]. The maintenance of EMT is governed by Sip1, which serves as a negative regulator of E-cadherin [5]. Importantly, when cancer cells finally colonize a niche, they undergo the reverse phenomenon, mesenchymal-epithelial transition (MET), which allows them to form a more stable connection between other cancer cells and the surrounding stroma [6].

Although the cross-talk between cancer cells invading the peritoneal cavity and residual PMCs is undoubtedly bidirectional, the impact of cancer cells on normal cells has not been systematically addressed. It was recently found that ovarian cancer cells may induce morphological (altered shape), molecular (altered intracellular organization), and functional (altered invasiveness) changes in the PMCs, called mesothelial-mesenchymal transition (MMT), being a form of EMT specific for mesothelial cells [7,8]. Unfortunately, these reports described less aggressive cancer histotypes and did not address some of the important mechanistic aspects of this phenomenon. Therefore, we designed a study in which we compared the ability of A2780 and SKOV-3 cells, which represent less aggressive endometrioid/clear cell histotypes, and of OVCAR-3 and primary EOCs, which are aggressive high-grade serous ovarian carcinomas (HGSOCs) [9], to induce MMT. Furthermore, we provided insight into the signaling, mediators, and cancer-promoting outcomes of MMT, with a particular emphasis on the molecular and functional variations between the less and more aggressive ovarian cancer histotypes.

## 2. Results

### 2.1. HGSOC Potentiates MMT in PMCs In Vitro and In Vivo

**Figure 1:**
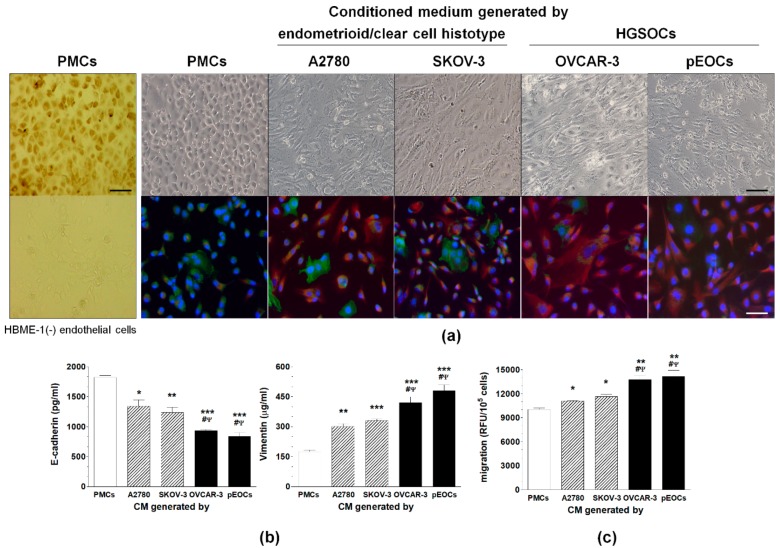
The induction of mesothelial-mesenchymal transition (MMT) in the peritoneal mesothelial cells (PMCs) exposed to autologous and cancer-derived conditioned medium (CM). (**a**) Representative pictures showing the development of spindle-shaped morphologies in the PMCs and confirmed by changes in E-cadherin (green) and vimentin (red) expression (the nuclei are stained blue). The very left panel of pictures shows the identification of PMCs according to the expression of HBME-1 antigen (brown cytoplasm). Below, HBME-1-negative endothelial cells (HMEC-1). Scale bars = 50 µm. (**b**) Changes in the E-cadherin and vimentin concentration in the cell lysates from the PMCs exposed to autologous and cancer-derived CM. (**c**) Migration of the PMCs undergoing the cancer-induced MMT. Experiments were performed with eight separate cultures of PMCs obtained from different donors, with eight separate cultures of primary epithelial cancer cells (pEOC), and with eight separate cultures of each established cancer cell line. The results are expressed as the mean ± SEM. *, *p* < 0.05 vs. PMCs; **, *p* < 0.01 vs. PMCs; ***, *p* < 0.001 vs. PMCs; ^#^, *p* < 0.05 vs. A2780; ^Ψ^, *p* < 0.05 vs. SKOV-3. RFU, relative fluorescence units.

Two lines of established ovarian cancer cells representing an endometrioid/clear cell histotype (A2780, SKOV-3), one established (OVCAR-3), and eight primary HGSOCs were used to generate conditioned medium (CM). The CMs were applied to PMCs for 10 days and induced signs of MMT, including a fibroblastic morphology, decreased expression of E-cadherin, increased expression of vimentin, and increased migration. The changes in the MMT-related proteins and the enhancement of PMC migration, were greater in response to the CM generated by the HGSOCs than in response to the CM from the A2780 and SKOV-3 cells (Figure 1).

**Figure 2:**
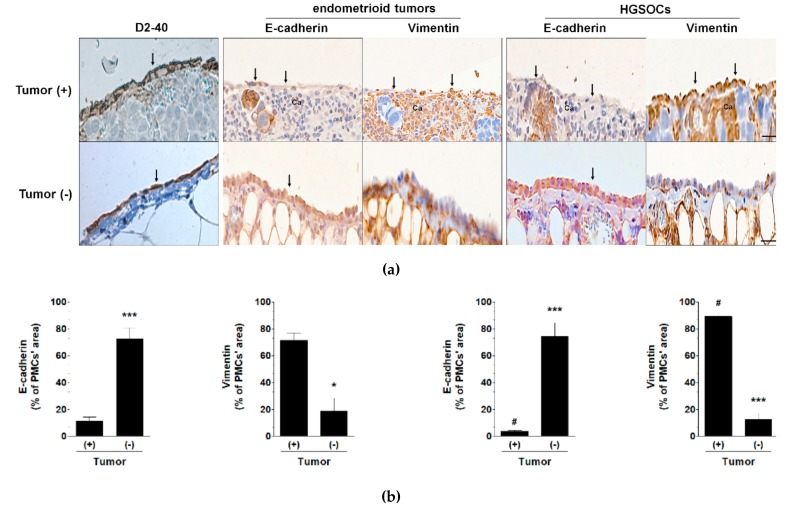
Features of MMT in human PMCs in vivo. (**a**) Expression of E-cadherin and vimentin in tumors from patients with high-grade serous ovarian cancer cells (HGSOCs) or endometrioid ovarian cancer, as well as in the cancer-free areas of the omentum from the same patients. The very left panel of pictures shows the identification of PMCs according to the expression of D2-40 antigen (brown reaction). Arrows indicate a layer of PMCs with different magnitudes of positive (brown) staining. Ca—cancer cells; Scale bars = 50 µm. (**b**) Quantification of the E-cadherin and vimentin levels in the biopsies using ImageJ software. Experiments were performed with tumors obtained from eight patients per group. The results are expressed as the mean ± SEM. ***, *p* < 0.001 vs. tumor (+); ^#^, *p* < 0.05 vs. endometrioid tumors.

The PMCs in tumors from patients with ovarian cancer exhibited lower expression of E-cadherin and higher expression of vimentin than the PMCs in the cancer-free zones of the omentum. In the PMCs from the patients with HGSOC, the expression of E-cadherin was decreased and the expression of vimentin was increased compared with the PMCs from the patients with endometrioid tumors (Figure 2).

**Figure 3:**
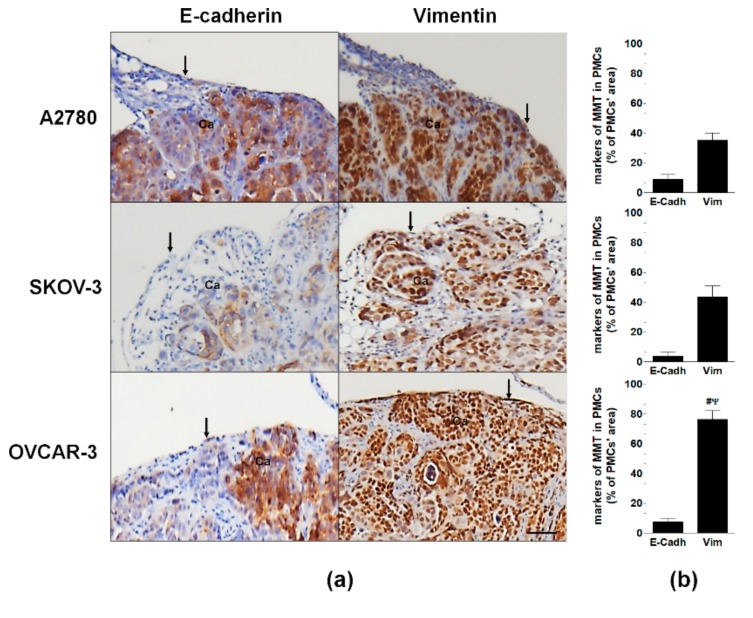
Features of MMT in murine PMCs in vivo. (**a**) Expression of E-cadherin and vimentin in xenografts that developed intraperitoneally following the injection of A2780, SKOV-3, or OVCAR-3 cells. Arrows indicate a layer of PMCs with different magnitudes of positive (brown) staining. Ca—cancer cells; Scale bars = 100 µm. (**b**) Quantification of the E-cadherin and vimentin levels in the biopsies using ImageJ software. Experiments were performed with tumors obtained from six animals per group. The results are expressed as the mean ± SEM. ^#^, *p* < 0.05 vs. A2780; ^Ψ^, *p* < 0.05 vs. SKOV-3.

A subsequent evaluation of the PMCs in xenografts that formed intraperitoneally in mice after the injection of A2780, SKOV-3, or OVCAR-3 cells showed that the expression of E-cadherin in tumors generated by OVCAR-3 cells was similar to that of tumors generated by A2780 and SKOV-3 cells, but the expression of vimentin in the OVCAR-3 xenografts was higher than that in the other two groups (Figure 3).

### 2.2. HGSOC-Driven MMT Involves Smad 2/3 and ILK

**Figure 4:**
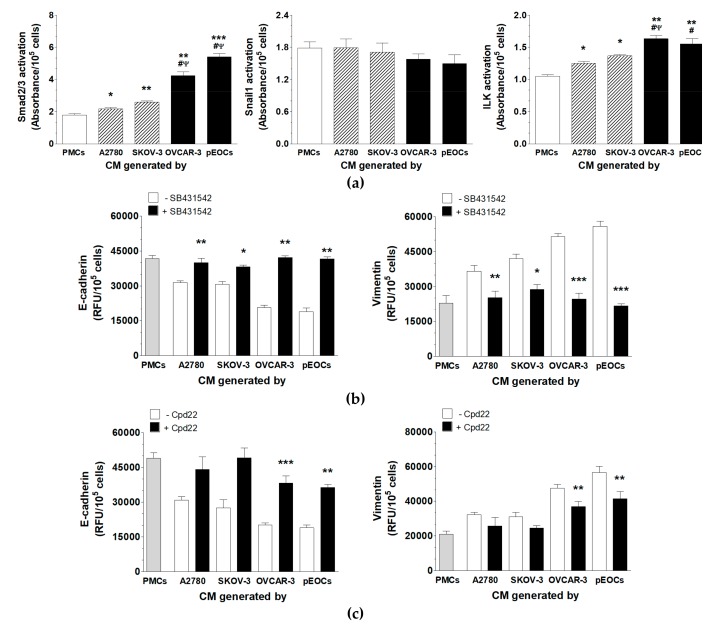
Signaling pathways involved in the cancer-induced MMT in PMCs. (**a**) Activation of Smad 2/3, Snail1, and ILK in the PMCs exposed to autologous and cancer-derived CM. Expression of E-cadherin and vimentin in the PMCs preincubated with chemical inhibitors of Smad 2/3 (SB431542) (**b**) and ILK (Cpd22) (**c**) prior to their exposure to autologous and cancer-derived CM. Experiments were performed with eight separate cultures of PMCs obtained from different donors, with eight separate cultures of primary epithelial cancer cells (pEOC), and with eight separate cultures of each established cancer cell line. Results are expressed as the mean ± SEM. *, *p* < 0.05 vs. PMCs; **, *p* < 0.01 vs. PMCs; ***, *p* < 0.001 vs. PMCs; ^#^, *p* < 0.05 vs. A2780; ^Ψ^, *p* < 0.05 vs. SKOV-3. RFU, relative fluorescence units.

Activation of Smad 2/3, Snail1 and ILK was analyzed in the PMCs exposed to autologous and cancer-derived CM. Experiments showed that CM from all cell types activated Smad 2/3 and ILK but failed to activate Snail1. The level of induction of both pathways was greater in response to CM from the HGSOCs than CM from the A2780 and SKOV-3 cells (Figure 4A).

To confirm that Smad 2/3 and ILK contributed to cancer-related MMT, PMCs were preincubated with chemical inhibitors of these pathways, and then the expression of E-cadherin and vimentin was retested. The inhibition of Smad 2/3, but not of ILK, led to the restoration of the E-cadherin and vimentin levels in the PMCs treated with CM from the A2780 and SKOV-3 cells. An analogous restoration of the MMT-associated proteins in PMCs that had been exposed to CM from the HGSOCs was observed upon the inhibition of both Smad 2/3 and ILK (Figure 4B,C).

### 2.3. HGSOC-Driven MMT Is Triggered by IGF-1, HGF, and TGF-β1

To evaluate the activators of the MMT released by cancer cells, the concentrations of EGF, HGF, IGF-1, and TGF-β1 were quantified. Comparative analysis revealed that the secretion of all tested proteins in the CM generated by the cancer cells was higher compared with the CM from the PMCs. Importantly, the release of HGF, IGF-1, and TGF-β1 by the HGSOCs was greater than by the A2780 and SKOV-3 cells (Table 1).

**Figure 5:**
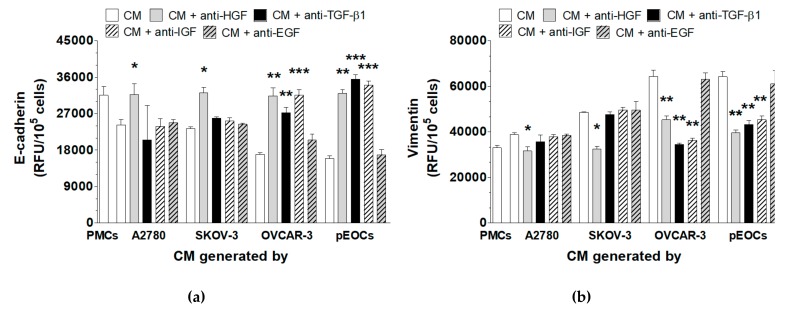
Identification of the cancer-derived mediators of MMT in PMCs. Expression of E-cadherin (**a**) and vimentin (**b**) in the PMCs whose exposure to autologous and cancer-derived CM was proceeded with a preincubation of the cancer-derived CM with specific neutralizing antibodies against HGF, TGF-β1, IGF-1, or EGF. Experiments were performed with eight separate cultures of PMCs obtained from different donors, with eight separate cultures of primary epithelial cancer cells (pEOC), and with eight separate cultures of each established cancer cell line. The results are expressed as the mean ± SEM. *, *p* < 0.05 vs. PMCs; **, *p* < 0.01 vs. PMCs; ***, *p* < 0.001 vs. PMCs. RFU, relative fluorescence units.

Experiments with neutralizing antibodies showed that it was possible to restore the levels of E-cadherin and vimentin to the control PMC levels when the CM from the A2780 and SKOV-3 cells was preincubated with the anti-HGF antibody. In the case of the CM produced by the HGSOCs, the restoration of both MMT-associated proteins was achieved upon the neutralization of HGF, IGF-1, and TGF-β1 (Figure 5).

### 2.4. PMCs Undergoing HGSOC-Induced MMT Are Strongly Tumor-Promoting

**Figure 6:**
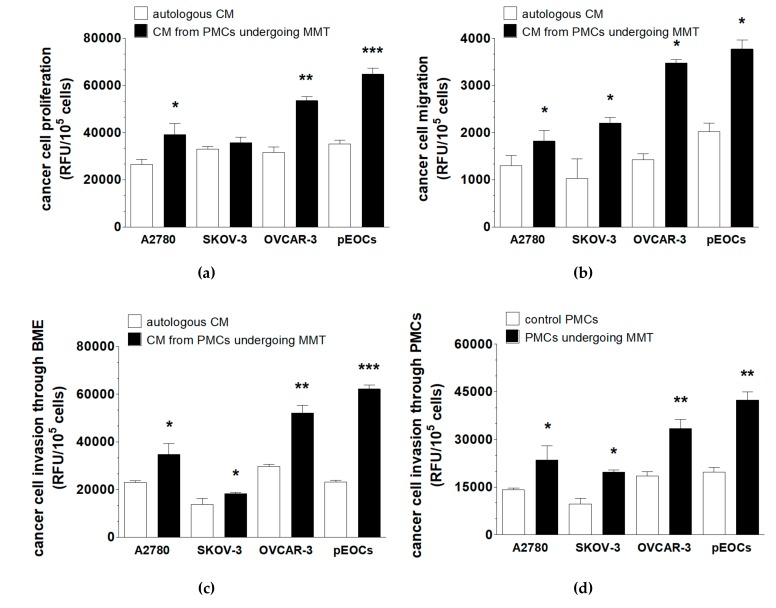
Analysis of the cancer-promoting effects of the PMCs undergoing MMT. Effect of the CM generated by cancer cells and PMCs in the MMT state on the proliferation (**a**), migration (**b**), invasion through BME (**c**), and invasion through PMCs (**d**) of the ovarian cancer cells. Experiments were performed with eight separate cultures of PMCs obtained from different donors, with eight separate cultures of primary epithelial cancer cells (pEOC), and with eight separate cultures of each established cancer cell line. The results are expressed as the mean ± SEM. *, *p* < 0.05, **, *p* < 0.01, ***, *p* < 0.001 vs. autologous CM or the control PMCs. RFU, relative fluorescence units.

Cancer cell-derived CM and CM from PMCs undergoing MMT were compared in regard to their effects on the proliferation, migration, and invasion (through a basement membrane extract (BME) and PMC monolayers) of cancer cells. The results showed that the CM from PMCs in the MMT state stimulated all of the tested phenomena more effectively than the CM from the cancer cells (Figure 6). The CM from the PMCs exposed to the HGSOCs promoted all aspects of cancer cell progression more strongly than the CM from the PMCs exposed to the A2780 and SKOV-3 cells (Figure 6).

Comparative analysis of the secretome of the PMCs, which focused on 10 soluble mediators of cancer cell progression, revealed that the CM from A2780 cells up-regulated the secretion of CCL2, CXCL12, HGF, and TGF-β1; the CM from SKOV-3 cells stimulated the secretion of CCL2, CXCL8, CXCL12, HGF, ICAM-1, TGF-β1, and VEGF; the CM from OVCAR-3 cells stimulated the secretion of CCL2, CXCL12, HGF, ICAM-1, PAI-1, TGF-β1, and VEGF; and the CM from primary EOCs stimulated the secretion of CCL2, CXCL8, CXCL12, HGF, ICAM-1, IL-6, PAI-1, TGF-β1, uPA, and VEGF. When the HGSOCs were compared with the A2780 and SKOV-3 cells, they appeared to more effectively support the production of all tested proteins (Table 2).

## 3. Discussion

Cancer cells modulate molecular and functional features of normal cells towards the development of a phenotype that supports the progression of the disease. Sandoval et al. showed that the conditioned medium from ovarian cancer cells induces MMT in PMCs, leading to their transformation into cancer-associated fibroblasts [7]. A similar observation was provided by Nakamura et al. with other cancer cell lines [8]. Notably, however, both reports are based on studies of cancer cells with relatively low (SKOV-3, A2780) or moderate (CaOV3) aggressiveness, whose genetic signatures classify them as having an endometrioid/clear cell (SKOV-3, A2780) and a serous (CaOV3) histotype [9]. These studies did not address the activity of high-grade serous ovarian cancer (HGSOC), which constitutes approximately 80% of EOCs [9] and contributes to two-thirds of all EOC-related deaths [10], making it the most common and the most lethal histotype. Indeed, HGSOCs are type II ovarian tumors that are characterized by a non-ovarian origin, rapid progression, and high aggressiveness, in contrast to the endometrioid, clear cell and low-grade serous histotypes, which are type I tumors that derive from the ovaries, are genetically stable and expand relatively slowly [11].

Having identified this gap and some other ambiguities regarding the mechanisms of cancer-derived MMT, we designed a comparative study in which the CM from type I ovarian cancers (A2780, SKOV-3) was compared with the CM from type II HGSOCs (an established OVCAR-3 cell line [9] and primary EOCs) for their ability to induce MMT. Moreover, we compared type I and II tumors with respect to the signaling pathways and cancer-derived mediators of this phenomenon, as well as potential cancer-promoting effects of PMCs in the MMT state. To our knowledge, this is the first report in which primary EOCs were used as a model of cancer-induced MMT, showing this process from a patient-oriented perspective.

Our experiments showed that the CM from type I and type II tumors effectively induced a PMC transformation that was consistent with MMT, that is, the acquisition of a fibroblast-like shape, decreased expression of E-cadherin, increased expression of vimentin, and increased migration. In contrast to recent papers in which similar features of PMCs in the MMT state were reported between the third and sixth days of exposure [7,8], we observed this phenotype after 10 days. This may be explained by some methodological differences, particularly the lower concentration of CM used (25% vs. 75% in [7]).

Importantly, all of the features that characterized MMT in the PMCs were observed to a higher degree in response to the HGSOCs than to the A2780 and SKOV-3 cells. This enhancement of MMT in vitro was also evident in vivo. The PMCs located near cancer cells in the tumors from patients with HGSOC displayed lower expression of E-cadherin and higher expression of vimentin than the PMCs from patients with endometrioid tumors. Others have reported the presence of PMCs in the MMT state in cancer patients using another marker of this phenotype (α-smooth muscle actin [7]), which provided important information about mechanisms by which cancer cells penetrate the mesothelial layer. Transcriptomic analysis performed during our project did not confirm, however, that PMCs undergoing the MMT display increased expression of this protein.

Because E-cadherin belongs to adherens junctions and is partly responsible for the integrity of PMCs, its deterioration may increase their penetrability by cancer cells. This challenges a theory that suggests that effective transmesothelial invasion requires the mechanical retraction of PMCs by cancer cells using forces generated by their myosin [12].

An enhancement of the MMT in PMCs, albeit restricted to increased vimentin expression, was observed in mice with OVCAR-3-generated xenografts compared with tumors generated by A2780 and SKOV-3 cells. Irrespective of the type of tumor cell injected, the layer of PMCs in the xenografts remained intact, as demonstrated using Wt1 staining [13] and was still present above the cancer cells that reached the stroma. This result conflicted with another study on mice in which cancer cell implantation within the peritoneum led to the incorporation of PMCs inside the tumor mass [8]. To some extent, the diverse locations of the PMCs may be explained by the different numbers of cells injected (2 × 10^6^ cells here vs. 1 × 10^6^ cells in [8]), different times of tumor development (2–3 weeks here vs. 3–4 weeks in [8]), and different types of cancer cells used (A2780, SKOV-3, OVCAR-3 here vs. A2780 and CaOV3 in [8]).

The increased ability of the HGSOCs to induce MMT implies that there must be some differences in the mechanism of this process between type I and type II tumors. To define them, we examined the signaling pathways contributing to MMT and the cancer-derived proteins that could be triggering the process. In regard to the signaling mechanisms, the HGSOCs induced MMT through Smad 2/3 and integrin-linked kinase (ILK), whereas the A2780- and SKOV-3-dependent processes exclusively involved Smad 2/3. In regard to Smad 2/3, our data agree with the study by Rynne-Vidal et al., who uncovered the role of this transcription factor in MMT provoked by SKOV-3 cells [14]. In fact, the Smad family of transcription factors, in particular Smad 2/3 interacting with the TGF-β1, are the molecules with the greatest significance for EMT in various models, including ovarian cancer [15].

The contribution of ILK to cancer-induced MMT has never been described before, although its involvement was reported as being critical for EMT in colorectal cancer [16], adenomyosis [17], and glomerulonephritis [18]. The most unexpected result is, however, that MMT elicited in the PMCs by both groups of cancers did not require Snail1. This is in contrast to the study by Sandoval et al., who found the up-regulation of Snail1 in PMCs forced to MMT by SKOV-3 cells [7]. Notably, they quantified the activation of Snail1 using qPCR. Here, we observed no activation of Snail1 at the protein level, which does not preclude the up-regulation of its mRNA. On the other hand, Snail1 is controlled at the transcriptional level by various molecules, including ILK [19], and one cannot rule out that despite its involvement in EMT of cancer cells [20], the induction of MMT in PMCs acts through ILK and Snail1 mRNA but bypasses Snail1 protein.

The discovery that ILK was needed for MMT in PMCs that was elicited by the HGSOCs, but not MMT that was elicited by the A2780 and SKOV-3 cells, may suggest that ILK activation may be important for the degree of the MMT that is induced by aggressive tumors. In fact, the activation of ILK is connected with the TGF-β1-Smad 2/3 pathway, as it is a downstream target of this signaling cascade [21]. This fact led us to search for a component of the cancer-derived CM that could activate MMT. We focused on four well-established EMT-inducing agents, EGF [22], HGF [23], IGF-1 [24], and TGF-β1 [25], whose secretion by the cancer cells was higher than that by the PMCs. Intervention studies showed that the HGSOCs triggered MMT using TGF-β1, HGF, and IGF-1, whereas the A2780 and SKOV-3 cells operated exclusively through HGF.

The identification of HGF as the sole mediator of MMT in the A2780 and SKOV-3 cells is consistent with the investigations by Nakamura et al., pointing to the role of this factor in the A2780- and CaOV3-dependent MMT [8]. Interestingly, when those authors compared the expression of HGF in tumors from patients with different EOC histotypes, the patients with serous tumors displayed higher expression of this factor than those with less aggressive tumors. On the other hand, the role of HGF in MMT has been questioned by Yu et al., who found that the treatment of PMCs with exogenous HGF prevented MMT induced by high glucose [26]. To some extent, these disparities may result from the concentration of HGF used in those studies (10–30 ng/mL) and in our studies (1–3 ng/mL).

However, the question remains as to why the HGSOC-induced MMT also involves, apart from HGF, IGF-1 and TGF-β1. We believe that it may depend on the concentration of these proteins in the CM. The levels of HGF, IGF-1, and TGF-β1 in the CM from the HGSOCs were considerably higher than those in the CM from the A2780 and SKOV-3 cells. This may indicate that there may be a threshold level that a given factor must reach to cause it to trigger MMT. In this regard Nakamura et al. found that the magnitude and reversibility of MMT differ between cell lines and depend on the concentration of HGF in cancer-derived CM [8].

The role of TGF-β1 in MMT has recently been demonstrated in PMCs treated with the CM from SKOV-3 cells [14]. This observation is inconsistent with our findings. Specifically, Sandoval et al. reported that PMCs treated with SKOV-3-derived CM acquire a morphology similar to that of cells exposed to 1 ng/mL of TGF-β1 [7]. Moreover, Rynne-Vidal et al. used exogenous TGF-β1 at an even higher dose of 4 ng/mL to examine the activation of the TGF-β1-Smad 3 pathway in PMCs exhibiting MMT [14]. In our opinion, it is likely that MMT in PMCs exposed to TGF-β1 in those papers could be considered an artifact because the CM from ovarian cancer cells has significantly lower levels of this factor (100–170 pg/mL in the CM from A2780/SKOV-3 cells and 300–450 pg/mL in the CM from HGSOCs). We think that similar to HGF [8], the level of TGF-β1 in the CM from A2780 and SKOV-3 cells was too low to induce MMT. At the same time, its concentration in the CM from HGSOCs probably reached a level that allowed the initiation of MMT.

The last part of our study concerned the analysis of the cancer-promoting activities of PMCs in the MMT state. Previous papers revealed that PMCs in the mesenchymal state stimulate adhesion and invasion of cancer cells, as well as angiogenesis and the formation of metastases [7,8,14]. Our study reinforced those findings by showing that CM from the PMCs that underwent MMT in response to HGSOCs promoted the proliferation, migration, and invasion of cancer cells more efficiently than the CM from PMCs in which MMT was triggered by less aggressive cancers. It is very plausible that this effect was associated with the higher secretion levels of several agents known to support ovarian cancer cell progression by these cells, such as CCL2, CXCL8, CXCL12, ICAM-1, IL-6, HGF, PAI-1, uPA, TGF-β1, and VEGF [2].

## 4. Materials and Methods

### 4.1. Chemicals

Unless otherwise stated, all chemicals and plastics were obtained from Merck (Darmstadt, Germany). An inhibitor of Smad 2/3 (SB 431542) was purchased from the Cayman Chemical Company (Ann Arbor, MI, USA); an inhibitor of ILK (Cpd22) was purchased from Merck; and antibodies against EGF (cat. no. AF236), HGF (cat. no. MAB294), IGF-1 (cat. no. AF-291-NA), and TGF-β1 (cat. no. AF-101-NA) were purchased from R&D Systems (Abingdon, UK).

### 4.2. Patients

The study included peritoneal metastases obtained from eight patients with HGSOCs and from eight patients with endometrioid tumors (stage IV). The tissues were fixed in formalin and then processed as described in [27]. Standard H+E staining was performed to identify cancer cells. Peritoneal mesothelium was identified according to positive staining for the D2-40 antigen (cat. no. IR072, Dako, Carpinteria, CA, USA). Antigen visualization was performed using Envision Flex (Dako). The procedures involving human subjects were in accordance with the Helsinki Declaration of 1975. All procedures, including the isolation of primary cancer cells, were approved by the Bioethics Committee at Poznan University of Medical Sciences (consent number 299/17), and all patients gave their informed consent.

### 4.3. Animal Studies

The experiments were performed with Scid mice (Charles River, Wilmington, MA, USA) that were injected i.p. with cancer cells, as described in [13]. The animals (six per group) were kept for 14 days and were then humanely sacrificed. Tumors were removed, fixed in formalin, dehydrated, embedded in paraffin, and sectioned. Then, the H+E staining and further immunocytochemistry were performed. Mesothelial cells were identified with immunostaining for the Wt1 antigen [28]. All tests were performed in compliance with the EU Directive 2010/63/EU and were approved by the Local Ethics Committee for Experiments on Animals in Poznan (consent number 32/2015).

### 4.4. Cell Cultures

Peritoneal mesothelial cells (PMCs) were isolated by enzymatic digestion of the omentum from eight patients undergoing abdominal surgery for noncancerous reasons. Briefly, the tissue was incubated in a solution of 0.05% trypsin and 0.02% EDTA for 20 min at 37 °C with gentle shaking. The cells were grown in medium M199 with L-glutamine (2 mM), penicillin (100 U/mL), streptomycin (100 µg/mL), hydrocortisone (0.4 µg/mL), and 10% fetal bovine serum (FBS) [29]. Cells were identified as pure mesothelial by their typical cobblestone appearance at confluence and uniform positive staining for HBME-1 antigen (Dako).

Ovarian cancer cells A2780 and SKOV-3 were obtained from the ECCC (Porton Down, UK) and propagated in RPMI 1640 medium with L-glutamine (2 mmol/L), penicillin (100 U/mL), streptomycin (100 g/mL), and 10% FBS. OVCAR-3 cells were purchased from the ATCC (Rockville, MD, USA) and grown in RPMI 1640 medium with L-glutamine (2 mmol/L), HEPES (10 mmol/L), sodium pyruvate (1 mmol/L), glucose (4500 mg/L), insulin (0.01 mg/mL), and 20% FBS [30]. The identity of these ovarian cancer cells was confirmed by analysis of NCBI database.

Primary EOCs were isolated from the tumors excised from eight patients with HGSOC. In brief, the tumors were carefully divided with a scalpel into eight-ten pieces of similar weight and then placed in a solution of trypsin and EDTA for 20 min at 37 °C with shaking. To confirm cancerous nature of the cells, they were resuspended in RPMI 1640 medium with 20% FBS and then they were identified using an immunofluorescence with antibodies against the epithelial-related antigen MOC-31 and CA125 (Abcam, Cambridge, UK). Lastly, ovarian cancer cells were cultured in RPMI 1640 supplemented with L-glutamine (2 mM) and 20% FBS [31]. The cells were identified as HGSOC according to a routine pathomorphological examination performed by a highly qualified specialist.

### 4.5. Conditioned Medium

To collect conditioned medium (CM), cells were seeded into 25 cm^2^ flasks and maintained until they reached 70–80% confluency. Then, they were washed, and fresh growth medium was added and incubated for 72 h to generate the CM. To collect serum-free CM for the immunoenzymatic assays, the cells were washed and serum starved for 72 h. Samples of CM were centrifuged, filtered, and stored at −80 °C until they were required. During the experiments, the PMCs were exposed to 25% autologous or cancer-derived CM diluted in the standard growth medium for 10 days.

### 4.6. Mesothelial-Mesenchymal Transition (MMT)

The concentrations of E-cadherin and vimentin in the cell lysates were examined using a Human E-Cadherin ELISA Kit and a Human Vimentin Profiling ELISA Kit (cat. no. 7771, Abcam, Cambridge, UK).

The expression of these proteins was also quantified with immunofluorescence. Cells were fixed in paraformaldehyde, washed, and treated overnight with antibodies against E-cadherin and vimentin (cat. no. ab15148 and ab16700, respectively, both from Abcam, 1:100). Then, the cells were washed and incubated with a DyLight 488 antibody (cat. no. ab96899, Abcam, 1:500) for 1 h. The fluorescence was recorded using a Synergy^TM^ 2 spectrofluorometer (BioTek Instruments, Winooski, VT). Representative staining was performed using a PathScan EMT Duplex IF kit (Cell Signaling Technology, Danvers, MA, USA) and visualized using an Axio Vert.A1 microscope (Carl-Zeiss, Jena, Germany).

As for the paraffin sections, immunohistochemistry was conducted using antibodies against E-cadherin and vimentin (cat. no. IR059 and IR630, respectively, Dako, 1:200). The staining was visualized using a Novolink Polymer Detection System (Novocastra Reagents, Wetzlar, Germany). The intensity of the staining was quantified using ImageJ v1.52a (http://rsb.info.nih.gov/ij/).

### 4.7. Signaling Pathways

Smad 2/3 was quantified using a PathScan^®^ Phospho-Smad2/Smad3 Sandwich ELISA kit (Cell Signaling). Activation of Snail1 was examined with a Phospho-SNAI1 Colorimetric Cell-Based ELISA Kit (Aviva Systems Biology, San Diego, CA, USA). ILK was quantified using a Human Integrin-Linked Kinase 1 (ILK-1) ELISA Kit (Abbexa, Cambridge, UK).

### 4.8. Cell Proliferation, Migration, and Invasion

Cancer cell proliferation in the presence of autologous CM or CM generated by PMCs in the MMT state was tested for 72 h using a Cell Proliferation Kit I (PromoKine; Heidelberg, Germany). Cancer cell migration through a polycarbonate membrane (8 μm pores) towards a chemotactic gradient generated by the two previously mentioned types of CM was examined for 4 h using ChemoTx chambers (Neuro Probe, Gaithersburg, MD, USA). The same assay was used to quantify the migration of PMCs undergoing an MMT. In that case, 1% FBS was used as a chemoattractant. Cancer cell invasion was measured with the Cultrex 96 Well BME Cell Invasion Assay (Trevigen Inc., Gaithersburg, MD, USA) using two protocols. First, cancer cells probed with calcein-AM were seeded into the upper part of the system (1 × 10^4^ cells/well) and allowed to invade the basement membrane extract (BME) towards a chemotactic gradient generated by autologous serum-free CM or CM produced by PMCs in the MMT state. In the second protocol, the control PMCs and PMCs undergoing an MMT (1 × 10^5^ cells/well) were seeded on the BME to form a monolayer. To ensure that the PMCs did not proliferate, they were serum-starved for 24 h. Then, ovarian cancer cells were added to the upper chamber on top of the PMCs. In this case, the invasion of the cancer cells was stimulated by 1% FBS. Irrespective of the invasion protocol used, the process was monitored for 24 h.

### 4.9. Cell Secretome

Concentrations of CCL2, CXCL8, CXCL12, HGF, ICAM-1, IL-6, PAI-1, TGF-β1, VEGF, and uPA were quantified using DuoSet^®^ Immunoassay Development kits (R&D Systems).

### 4.10. Intervention Studies

In some experiments, the expression of E-cadherin and vimentin was examined in PMCs that were preincubated for 4 h with inhibitors of Smad 2/3 (SB 431542; 200 nM) and ILK (Cpd22; 1000 nM) before being exposed to autologous or cancer-derived CM. In other experiments, the expression of these proteins was tested in the presence of autologous or cancer-derived CM that was preincubated for 72 h with antibodies neutralizing EGF (30 ng/mL), HGF (1 µg/mL), IGF-1 (200 ng/mL), and TGF-β1 (400 ng/mL). The specificity and effectiveness of the inhibition/neutralization were verified during preliminary studies in which either the concentrations or times of exposure were optimized.

### 4.11. Statistics

Statistical analysis was conducted using GraphPad Prism™ 7.00 (GraphPad Software, San Diego, CA, USA). Since all variables were non-parametric, the means were compared with the Wilcoxon test. In particular, HGSOCs (primary EOCs and OVCAR-3 cells) were compared separately with PMCs, A2780 cells, and SKOV-3 cells. Individual marks of statistical significance have been explained in figure legends. The results were expressed as the mean ± SEM. Differences with a *p* value < 0.05 were considered to be statistically significant.

## 5. Conclusions

Collectively, our findings showed that HGSOCs have a greater potential than less aggressive type I ovarian tumors to trigger MMT in PMCs; this was associated with different signaling routes and mediators engaged in this process and with more pronounced cancer-promoting outcomes. Taking this into account, the unique pattern of MMT initiated by the HGSOCs in PMCs may be considered a separate factor responsible for the high aggressiveness of this type of ovarian malignancy. Therefore, particular attention should be directed towards the therapeutic targeting of this phenomenon, especially having in mind that malignant ascites obtained from patients with HGSOCs have been found to trigger EMT in ovarian cancer cells, increasing their invasive capabilities [32]. To strengthen these finding, further in vivo experiments seem to be required in which the development of peritoneal tumors will be monitored upon the co-injection of cancer cells with differentiated PMCs. 

## Figures and Tables

**Table 1:** Concentration of the proteins known to induce epithelial-mesenchymal transition (EMT) in the conditioned medium harvested from PMCs and ovarian cancer cells.

Soluble Agent (pg/mL)	PMCs	A2780	SKOV-3	OVCAR-3	pEOCs
EGF	40 ± 2	48 ± 1 *	39 ± 1	53 ± 1 *^Ψ^	46 ± 2 *^Ψ^
HGF	1195 ± 20 *	1342 ± 23 *	1552 ± 21 **	2704 ± 42 ***^#^^Ψ^	3055 ± 56 ***^#^^Ψ^
IGF-1	134 ± 3	145 ± 2 *	151 ± 8 *	296 ± 32 **^#^^Ψ^	491 ± 16 ***^#^^Ψ^
TGF-β1	114 ± 11	167 ± 5 *	151 ± 8 *	311 ± 7 ***^#^^Ψ^	434 ± 12 ***^#^^Ψ^

Measurements were performed using six cultures of PMCs isolated from different donors and with six separate cultures of cancer cells. The results are expressed as the mean ± SEM. *, *p* < 0.05 vs. PMCs; **, *p* < 0.01 vs. PMCs; ***, *p* < 0.001 vs. PMCs; ^#^, *p* < 0.05 vs. A2780; ^Ψ^, *p* < 0.05 vs. SKOV-3; pEOCs, primary epithelial ovarian cancer cells.

**Table 2:** Production of soluble proteins known to contribute to various aspects of ovarian cancer cell progression by the PMCs exposed to autologous and cancer-derived conditioned medium.

Soluble Agent (pg/10^5^ Cells)	PMCs	A2780	SKOV-3	OVCAR-3	pEOCs
CCL2	246 ± 4	288 ± 2 *	288 ± 1 *	386 ± 4 *^#^^Ψ^	420 ± 42 **^#^^Ψ^
CXCL8	291 ± 12	245 ± 34	306 ± 8 *	278 ± 12	422 ± 6 ***^#^^Ψ^
CXCL12	171 ± 8	205 ± 9 **	223 ± 10*	385 ± 21 **^#^^Ψ^	419 ± 18 ***^#^^Ψ^
HGF	33 ± 4	170 ± 11 ***	63 ± 7 *	223 ± 7 ***^#^^Ψ^	202 ± 10 ***^#^^Ψ^
ICAM-1	98 ± 2	82 ± 4	148 ± 1 *	271 ± 16 ***^#^^Ψ^	334 ± 3 ***^#^^Ψ^
IL-6	75 ± 2	73 ± 4	69 ± 6	81 ± 4	221 ± 2 ***^#^^Ψ^
PAI-1	1932 ± 32	2019 ± 63	1899 ± 28	2321 ± 1 *^#^^Ψ^	4103 ± 92 ***^#^^Ψ^
TGF-β1	127 ± 1	148 ± 1 *	163 ± 2 *	311 ± 22 ***^#^^Ψ^	380 ± 1 ***^#^^Ψ^
uPA	6 ± 1	7 ± 1	8 ± 1	7 ± 1	18 ± 1 **^#^^Ψ^
VEGF	227 ± 4	225 ± 11	262 ± 12 *	287 ± 18 *^#^	454 ± 4 ***^#^^Ψ^

Measurements were performed using the conditioned media generated by eight cultures of PMCs isolated from different donors and by eight separate cultures of cancer cells. The results are expressed as the mean ± SEM. *, *p* < 0.05 vs. PMCs; **, *p* < 0.01 vs. PMCs; ***, *p* <0.001 vs. PMCs; ^#^, *p* < 0.05 vs. A2780; ^Ψ^, *p* < 0.05 vs. SKOV-3. pEOCs, primary epithelial ovarian cancer cells.
